# An Automated Fluorescence Microscopy-Based Sensing System for Continuous Detection of Airborne Asbestos Fibers on a PM_2.5_ Monitoring Platform

**DOI:** 10.3390/s26103163

**Published:** 2026-05-16

**Authors:** Akio Kuroda, Kenichiro Kaga, Tomoki Nishimura, Kyoka Ichikawa, Shogo Yamazaki, Hisakage Funabashi, Takeshi Ikeda, Takenori Ishida

**Affiliations:** 1 Graduate School of Integrated Sciences for Life, Hiroshima University, 1-3-1 Kagamiyama, Higashi-Hiroshima 739-8530, Hiroshima, Japan; tn-nishimurat@hiroshima-u.ac.jp (T.N.); d245601@hiroshima-u.ac.jp (K.I.); d264652@hiroshima-u.ac.jp (S.Y.); hisafuna@hiroshima-u.ac.jp (H.F.); ikedatakeshi@hiroshima-u.ac.jp (T.I.); tishishi@hiroshima-u.ac.jp (T.I.); 2 DKK-TOA Corporation, 613 Kitairiso, Sayama 350-1388, Japan; k-kaga@toadkk.co.jp

**Keywords:** airborne asbestos sensing, fluorescence microscopy, automated monitoring, AI-assisted image analysis, PM_2.5_ monitoring platform

## Abstract

**Highlights:**

**What are the main findings?**

- A fully automated system that integrates air sampling, fluorescent staining, fluorescence microscopy, and AI-assisted fiber recognition was developed for detecting airborne asbestos fibers.
- Automated measurements can be completed within a 30-min cycle (20 min for sampling and approximately 10 min for staining and detection) without manual microscopic observation.

**What are the implications of the main findings?**

- Conventional methods relying on manual sampling and electron microscopy are complex and time-consuming, hindering continuous automated monitoring of airborne asbestos.
- The novel approach provides a practical framework for the environmental surveillance of airborne asbestos and marks significant progress toward next-generation automated asbestos monitoring systems.

**Abstract:**

Despite regulations restricting asbestos use in many developed countries, asbestos-containing materials (ACMs) persist in aging buildings and can release airborne fibers during demolition and renovation. Therefore, continuous monitoring of airborne asbestos fibers is essential for environmental safety and exposure assessment. Fluorescence microscopy (FM) with fluorescently labeled asbestos-binding proteins offers greater sensitivity and selectivity in detection compared with conventional phase contrast microscopy (PCM). However, its practical application is limited by manual sample preparation and microscopic observations. This study introduces the conceptual design and initial development of an automated FM-based sensing system for monitoring airborne asbestos fibers. The system was constructed by modifying a commercial PM_2.5_ continuous air sampling platform and integrating automated fluorescent staining, FM imaging, and AI-assisted image analysis for fiber recognition and counting. The system automatically reports airborne asbestos concentrations with corresponding fluorescence images and advances the membrane filter to enable continuous measurements. Performance evaluation using pulverized ACMs showed an overall agreement within 14.1% with PCM–scanning electron microscopy measurements at the group level. Although variability was observed at low fiber concentrations owing to stochastic sampling effects, the results validate the feasibility of automated FM-based sensing for continuous environmental monitoring of airborne asbestos fibers.

## 1. Introduction

Continuous monitoring of airborne asbestos fibers is essential for ensuring environmental safety, particularly at demolition sites involving asbestos-containing materials (ACMs) [1,2,3]. Asbestos refers to a group of six naturally occurring fibrous silicate minerals—chrysotile and five amphiboles (amosite, crocidolite, actinolite, tremolite, and anthophyllite)—that were extensively utilized in construction materials. When the structural integrity of ACMs is compromised, asbestos fibers can be released into the air. Inhalation of these fibers presents significant health risks, including pleural mesothelioma and lung cancer [4,5,6]. Although the use of asbestos has been banned in many developed countries, ACMs persist in older buildings, and their demolition substantially increases the risk of fiber release [7,8], thereby contributing to ongoing cases of asbestos-related diseases. Consequently, continuous monitoring of airborne asbestos fiber concentrations and the prompt detection of any leakage are crucial for environmental protection. However, to the best of our knowledge, there have been no prior reports of a fully automated system capable of continuously detecting airborne asbestos fibers.

Generally, airborne asbestos fibers are measured by collecting airborne particles and fibers on membrane filters. The filters are then rendered transparent with acetone, and fibers are counted using phase contrast microscopy (PCM) based on established criteria: fibers longer than 5 µm, thinner than 3 µm, and with aspect ratios greater than 3:1 [9]. However, a significant limitation of PCM is its inability to differentiate between asbestos and non-asbestos fibers. This limitation can result in the inadvertent inclusion of non-asbestos fibers in the count, leading to overestimation and highlighting the need for differential identification of asbestos fibers. In Japan, any PCM measurement exceeding 1 fiber per liter (f/L) is subject to re-validation using scanning electron microscopy (SEM) and energy-dispersive X-ray spectroscopy [10]. In the United States, when significant airborne fiber contamination is detected in a sample, subsequent validation using transmission electron microscopy (TEM) is preferred [11]. However, owing to the high-cost and time-consuming asbestos analyses, comprehensive monitoring using this technique is generally impractical for routine use at demolition sites.

To address the limitations of electron microscopy in on-site asbestos detection, fluorescent probes based on asbestos-binding proteins have been developed [12,13,14]. Two types of fluorescent reagents—one specific to chrysotile [12] and another to amphibole asbestos [13]—enable the detection of all asbestos types. Importantly, these probes do not bind to nine common non-asbestos fibrous materials, including man-made asbestos substitutes such as glass fibers and Rockwool, which frequently interfere with PCM analysis [14]. Consequently, the probes can effectively distinguish asbestos from non-asbestos fibrous materials, with the exception of silicon carbide whiskers [14]. Asbestos fibers collected on a membrane filter can be fluorescently labeled within minutes, allowing for rapid visualization under fluorescence microscopy (FM) [14,15]. Owing to its potential for the rapid, on-site detection of airborne asbestos, the FM-based method has been incorporated into Japan’s Asbestos monitoring manual as a screening tool for detecting asbestos leakage at demolition sites [10]. In parallel, automated image analysis and fiber counting methods have also been developed for FM-based asbestos detection. Early fluorescence-based fiber-counting approaches [16] and more advanced AI-based fiber recognition using deep learning [17] have been reported. In addition, a dual-mode high-throughput microscopy system that combines automated image acquisition with fiber enumeration using ImageJ software has been developed [18]. However, these studies primarily focused on individual elements of the detection process, such as imaging, image analysis, or probe specificity.

A major challenge in the continuous monitoring of airborne asbestos fiber concentrations is the selection of an appropriate base device to integrate the individual elements and automate the FM method. One promising solution is the use of a PM_2.5_ automatic detector, which continuously collects airborne particulates on a tape-like membrane filter and determines PM_2.5_ concentrations by irradiating the accumulated particles with beta rays and analyzing their absorption levels [19]. This detector features a reel unit that securely holds the tape-like membrane filter as the pump unit draws in air for sampling. Following the measurement of PM_2.5_ particles, the reel unit advances the tape-like membrane filter, exposing a fresh section for subsequent sampling. Originally designed for continuous, onsite environmental PM_2.5_ monitoring, this system provides an optimal platform for the integration of FM-based asbestos detection.

In this study, we developed an automated FM-based sensing system by incorporating a fluorescent staining unit, an FM unit, and AI-assisted fiber counting software into a modified commercial PM_2.5_ monitoring platform. The performance of the proposed system was evaluated by comparing it with conventional manual sampling and counting methods, specifically PCM combined with SEM. This approach establishes a practical framework for the environmental surveillance of airborne asbestos and represents a significant advancement toward the development of next-generation automated asbestos monitoring systems.

## 2. Materials and Methods

### 2.1. Materials

Chrysotile (Zimbabwe), amosite (Transvaal), and crocidolite (Cape) were sourced from the Japan Association for the Study of Fiber Materials (Atsugi, Japan). ACMs, including two types of calcium silicate boards and a slate board, were obtained from F&A Technology (Osaka, Japan). One calcium silicate board contained 6.6% amosite, 4.1% crocidolite, and 2.8% chrysotile, whereas the other board contained 24% amosite and 0.9% chrysotile. The slate board contained 7.4% chrysotile. An asbestos-free calcium silicate board was procured from Sekisui Chemical Co., Ltd. (Tokyo, Japan). The asbestos fluorescent probe (Asbester Air2), which comprises high-affinity asbestos-binding proteins labeled with fluorescent dyes for the detection of all asbestos types, was supplied by Siliconbio Inc. (Hiroshima, Japan). Asbestos fibers do not display intrinsic fluorescence under visible light excitation; however, after staining with Asbester Air2, they become readily detectable under FM.

### 2.2. An Automated System for Detecting Airborne Asbestos Fibers

**Figure 1:**
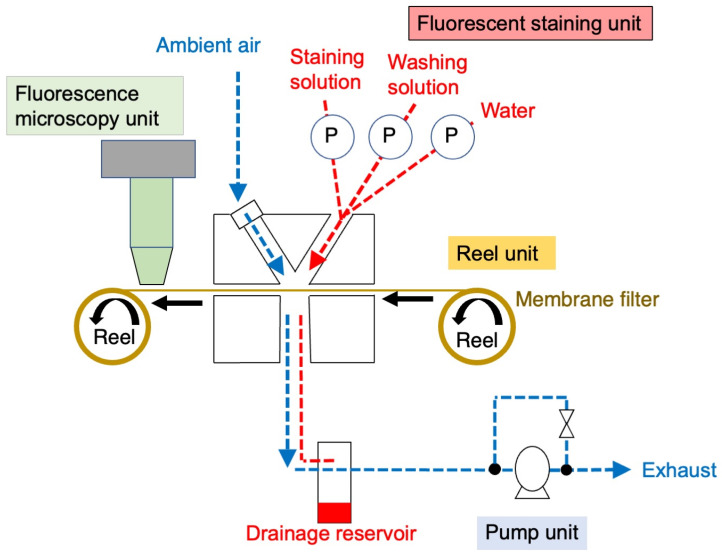
System architecture of the automated fluorescence microscopy (FM)-based sensing system for continuous monitoring of airborne asbestos fibers. The system comprises reel and pump units adapted from a commercial PM_2.5_ continuous air sampling and monitoring platform, alongside newly designed fluorescent staining and FM units. P denotes a roller-tube peristaltic pump, whereas the blue and red dotted lines represent the directions of air and reagent flows, respectively.

An automated system for detecting airborne asbestos fibers was developed by modifying the reel and pump units of a commercial PM_2.5_ continuous monitoring detector (FPM-377C, TOA-DKK Co., Ltd., Tokyo, Japan). The modified system was further equipped with a fluorescent staining unit, an FM unit, AI-assisted fiber counting software, and a central control system (Figure 1).

#### 2.2.1. Reel and Pump Units

The reel and pump units of the commercial PM_2.5_ automatic detector were designed to collect ambient air samples onto a tape-like membrane filter, advancing the membrane to expose a fresh sampling area following each PM_2.5_ measurement. To prevent air leakage, the reel unit clamped the tape-like membrane filter during air sampling. Upon completion of the PM_2.5_ measurement, the clamp was released, and the membrane filter was advanced by the reel mechanism. For adaptation to an automated system for airborne asbestos fiber detection, several modifications were implemented. The original Teflon tape-like filter was replaced with a cellulose mixed ester membrane, which is used for standard asbestos sampling. The beta-ray source, previously located above the clamp, was removed and substituted with a newly developed fluorescent staining unit. Following air sampling by the pump unit, fluorescent staining reagents were sequentially applied to the sampled area (15 mm in diameter) of the tape-like membrane filter. Excess liquid was removed by suction through the pump, with a drain tank installed upstream to prevent liquid ingress into the pump system. After staining, the clamp was released, and the tape-like membrane filter was advanced, positioning the stained sampling area beneath the objective lens of the FM unit.

#### 2.2.2. Fluorescent Staining Unit

The fluorescent staining unit was designed to automate the staining of asbestos fibers on the membrane filter, following established manual protocols [15]. Three roller-tube peristaltic pumps supplied each reagent from separate reservoirs containing fluorescent staining reagent, washing solution, and rinsing water. Each reagent was dispensed through an independent nozzle to prevent cross-contamination. Upon completion of the staining process, the reel unit released the tape-like membrane filter, advancing it approximately 40 mm to position the stained sampling area directly beneath the objective lens of the FM unit. A signal indicating filter advancement was transmitted via an I/O terminal to Captomator software version 1.0.0.104 (Mitani Corporation, Tokyo, Japan), which subsequently triggered the FM unit to initiate image acquisition.

#### 2.2.3. Fluorescence Microscopy Unit

The FM unit was equipped with an LED excitation light source (470 nm, Mightex LCS-0470-03-22), a 40× objective lens (NA = 0.6), a long-pass filter set (Excitation Filter: 475 AF40, Dichroic Mirror: 505DRLP, Fluorescence Filter: 510ALP, Omega Optical LLC, Brattleboro, VT, USA), and a 1-inch color CMOS image sensor (MIchrome20, Tucsen Photonics, Fuzhou, China). To maintain the flatness of the membrane filter during imaging, a support structure (27 mm wide, 30 mm long) was installed beneath the objective lens. A focus stacking technique [20] was employed to generate a single composite image (660 µm × 440 µm) with an extended depth of field by combining multiple images acquired at different focal planes. This process was automated using a z-axis stage (ALV-600B-HOM, Chuo Precision Industrial Co., Tokyo, Japan) under the control of the Captomator software (Mitani Corporation). During focus stacking, the z-axis was incrementally advanced in 2 µm steps, capturing a total of 25 images. The resulting focus-stacked images were automatically saved to a designated folder within the AI-assisted fiber-counting software.

#### 2.2.4. AI-Assisted Fiber-Counting Software

Image analysis and fiber counting were conducted automatically using an AI-based object detection model built on the YOLOv4 framework and trained with fluorescence images of asbestos fibers, as previously described [17]. Notably, the optical configuration of the current automated system differs from that of the previous setup. To retrain the YOLOv4-based detection model, a new training dataset comprising 1123 fluorescence images was generated from pulverized ACMs dispersed on membrane filters using the present optical configuration. Fibers exceeding 5 µm in length, less than 3 µm in width, and with an aspect ratio exceeding 3:1 were manually annotated as asbestos fibers. Of these, 994 images (88.5%) were used for training, 110 images (9.8%) for validation, and 19 images (1.7%) for testing. The dataset was split at the image level, while ensuring that samples containing different asbestos types (chrysotile, amosite, and crocidolite) were included in each subset. In accordance with standard asbestos measurement protocols, the AI model was also trained to exclude aggregated fibers [10]. This retraining process ensured that the detection model was optimized for the image characteristics produced by the current optical system.

The software package, “Asbestos Inspection,” incorporates AI-assisted asbestos recognition and fiber counting based on the YOLOv4 deep learning model. The program continuously monitors a designated folder for newly saved focus-stacked images and automatically analyzes them to detect asbestos fibers. Detection results are displayed alongside the processed image, number of detected fibers, and calculated airborne asbestos concentration (fibers/L). The airborne asbestos fiber concentration was determined by converting the number of fibers counted on the membrane filter to a concentration per unit volume of sampled air as follows:

*C* (f/L) = *A* × *N*/(*a* × *n* × *V*), (1)

where

*C* (f/L) represents the airborne asbestos fiber concentration (fibers per liter of air), *A* (mm^2^) represents the effective area of the membrane filter (a circular area with a diameter of 15 mm), *N* represents the number of asbestos fibers counted in the observed fields, *a* (mm^2^) represents the area of a single microscopic field of view (0.66 mm × 0.44 mm), *n* represents the number of fields counted, and *V* (L) represents the total volume of air sampled.

#### 2.2.5. Central Management Software

The central management software, “Asbestos Host Program,” oversees and controls the entire system. This allows for independent operation of each unit, including the pump, reel, fluorescent staining, and FM modules, while also coordinating their activities according to pre-configured sequences. A flowchart of the control logic and operational sequence of the software is shown in Supplementary Figure S1. Users can configure parameters, such as the sampling duration, directly through the Asbestos Host Program.

### 2.3. Chamber Experiment

The performance of the automated system was evaluated using a 400-L chamber specifically designed for asbestos dispersion, with technical support provided by F&A technology (Osaka, Japan). Airborne particles were generated by introducing asbestos fibers or pulverized ACM samples into a glass cylinder and subsequently dispersing the resulting particles into the chamber. A mixing fan ensured uniform particle distribution throughout the chamber, whereas a dust monitor continuously measured particle concentration. An exhaust pump was employed to reduce particle concentrations as needed. Both the automated system and a manual sampling setup were connected to the chamber via sampling tubes, enabling simultaneous measurements.

### 2.4. Statistical Analysis

To evaluate reproducibility and agreement between the automated and manual methods, repeated measurements were compared using Bland–Altman analysis [21]. The limits of agreement were defined as the mean difference ± 1.96 standard deviations (SD), representing the 95% limits of agreement. This analysis provided a quantitative evaluation of the concordance between the two methods across the entire measurement range.

## 3. Results

### 3.1. Development of an Automated System for Detecting Airborne Asbestos Fibers

We developed an automated FM-based sensing system for the continuous detection of airborne asbestos fibers, integrated onto a PM_2.5_ monitoring platform (Figure 1).

**Figure 2:**
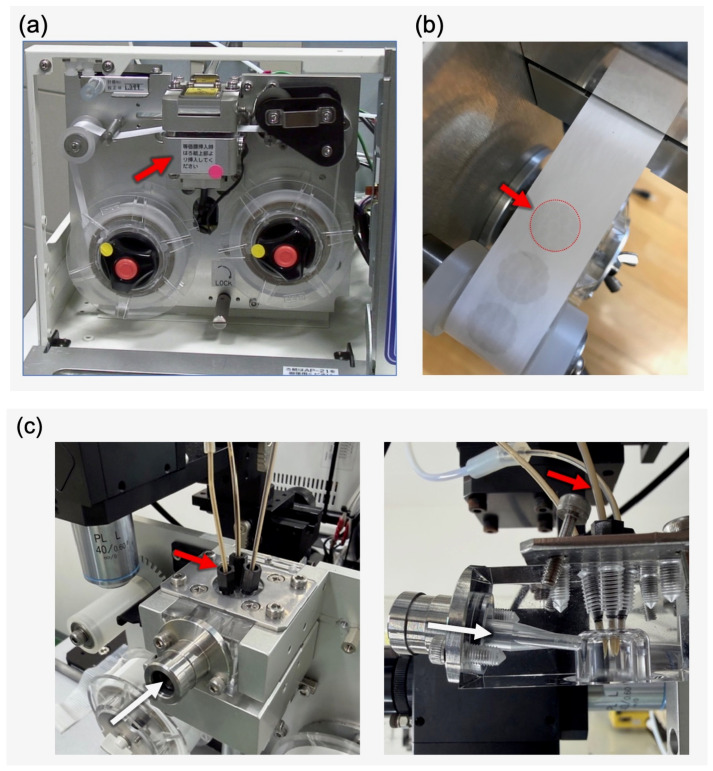
Reel unit of the PM_2.5_ detector, membrane collection spot, and fluorescent staining unit. (**a**) Prior to air sampling, the bottom support (red arrow) moves upward to clamp the tape-like membrane filter between the bottom and top supports. Upon completion of air sampling, the bottom support moves downward, enabling the reel unit to advance the membrane filter leftward and expose a fresh sampling area. (**b**) Airborne particles are collected on a designated collection spot (red) of the tape-like membrane filter. (**c**) Newly designed air gate and three independent nozzles (red arrow) for sequential reagent delivery are installed above the clamp of the reel unit. The right panel displays the interior of the clamp, with white arrows indicating the direction of airflow.

**Figure 3:**
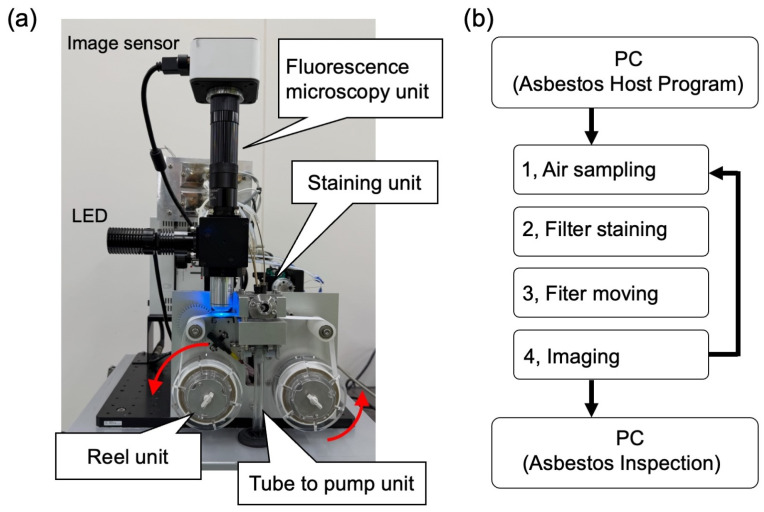
Overview of the automated system (**a**) and workflow of the continuous detection of airborne asbestos fibers (**b**). The red arrows in (**a**) indicate the direction of rotation of the reel. In (**b**), the vertical arrows indicate the sequential flow of the measurement process, and the right-side arrow indicates repetition of the measurement cycle from imaging back to air sampling.

The PM_2.5_ automatic detector continuously collects airborne particles onto a tape-like membrane filter while simultaneously measuring PM_2.5_ concentrations [19]. During air sampling, the reel unit clamped the tape-like membrane filter, preventing air leakage and ensuring efficient particle collection (Figure 2a). Upon completion of PM_2.5_ measurements, the reel unit released the tape-like filter and advanced it, winding the sampled section onto the take-up reel (Figure 2b). To enable automated detection of airborne asbestos fibers, several modifications were made to the reel and pump units of the original PM_2.5_ detector: (i) the Teflon filter was replaced with a cellulose mixed ester tape-like membrane filter; (ii) the beta-ray source, previously located above the clamp, was substituted with a newly designed air gate and three independent nozzles for the sequential application of fluorescent staining reagent, washing solution, and rinsing water (Figure 2c); and (iii) a drainage reservoir was installed immediately upstream of the pump. The modified system was further equipped with dedicated fluorescent staining and FM units (Figure 3a), all managed by a central management software, the “Asbestos Host Program.”

Initiating the sampling process via the “start measurement button” on the Asbestos Host Program activates the pump unit. Airborne particles were collected from the designated collection spot of the tape-like membrane filter. After the specified sampling duration, the fluorescent staining unit operated automatically, first dispensing a washing solution onto the membrane filter, then applying the fluorescent staining reagent to label the asbestos fibers, and subsequently performing additional washing and rinsing steps to remove excess staining solution. Upon completion of the staining process, the reel unit released the membrane filter, allowing it to be wound onto the take-up reel. The stained area was then positioned directly beneath the objective lens of the FM unit, where excitation light was applied, and images were captured and analyzed using Asbestos Inspection software. This sequence was repeated until the Asbestos Host Program was stopped (Figure 3b).

### 3.2. Image Capture Using a Focus Stacking Technique

The cellulose membrane filter displayed a porous architecture composed of intricately entangled cellulose fibers, resulting in a microscopically uneven surface with multiple height variations. Standard protocols for asbestos PCM analysis specify that multiple focal planes must be examined by vertically scanning through the sample depth, typically within a range of approximately 10–15 µm from the upper surface of the filter [22]. Capturing images at a single focal plane limited the ability to assemble all particles and fibers within the field of view into simultaneous focus, resulting in some fluorescently stained asbestos fibers remaining out of focus.

To address these depth-of-field limitations, a focus stacking technique [20] was implemented. This technique generates a single, highly focused composite image by combining 25 images acquired at different focal planes at 2-µm intervals along the z-axis. Once the image set is saved in the designated folder, the Asbestos Inspection software initiates the AI-assisted fiber-counting process and displays the results. Each measurement cycle—including 20 min of sampling at 10 L/min and approximately 10 min of staining and detection—lasted approximately 30 min. Under these sampling conditions, the detection of a single fiber corresponded to an airborne concentration of 3.0 fibers/L.

### 3.3. Improvement of AI-Assisted Fiber-Counting Software

Previously, 176 fluorescent images (111 containing asbestos fibers and non-asbestos fluorescent particles and 65 containing only fluorescent particles) were obtained from 13 airborne dust samples and used to train the YOLOv4 model [17]. However, the optical configuration of the current automated system, including the camera, objective lens, and imaging parameters, differs from that utilized in the previous configuration, rendering the earlier trained model incompatible. To address this issue, a new training dataset comprising 1123 fluorescence images was generated by pulverizing ACMs and non-asbestos building materials, followed by image acquisition using the present optical system. Each image was manually annotated to identify asbestos fibers, and the dataset was used to retrain the YOLOv4-based detection model. This retraining enabled accurate fiber recognition under the specific imaging conditions of the present system. The number of annotated asbestos fibers was 6493 in the training set (average 6.5 fibers/image), 1341 in the validation set (average 12.2 fibers/image), and 112 in the test set (average 5.9 fibers/image). The number of annotated non-fibrous particles was 85,705 in the training set (average 86.2 particles/image), 12,396 in the validation set (average 112.7 particles/image), and 2457 in the test set (average 129.3 particles/image). Despite these improvements, some false-positive detections persisted, primarily resulting from the misidentification of non-fibrous fluorescent particles and the erroneous classification of closely spaced particles as asbestos fibers.

**Figure 4:**
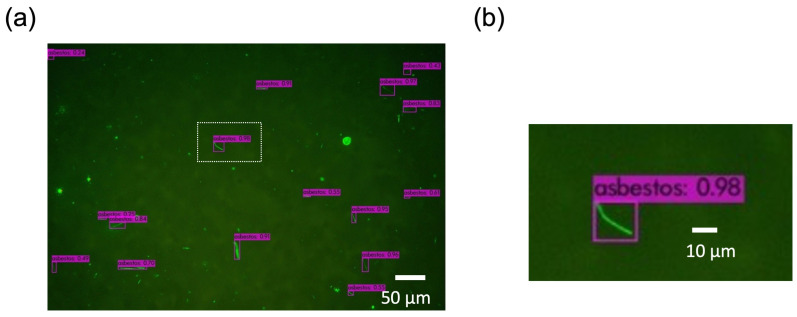
Image analysis by Asbestos Inspection software. (**a**) Fibers detected by the YOLOv4-based deep learning model are highlighted with bounding boxes (red and purple) and corresponding confidence scores (maximum = 1.00). (**b**) Magnified view of the white boxed area in (**a**).

To mitigate these false positives, a confidence score threshold of 0.32 was implemented, which effectively minimized false positives while maintaining high performance metrics: an accuracy of 0.991 ± 0.012, recall of 0.902 ± 0.062, precision of 0.902 ± 0.111, and an F-score of 0.902 ± 0.056 (Supplementary Table S1). These results represent a slight improvement over previous values for recall (0.898), precision (0.898), and F-score (0.898), with a marginal decrease in accuracy compared with the previous value of 0.997 [17]. When a fluorescent fiber was recognized as asbestos by the YOLOv4-based deep learning model, it was highlighted with a red-purple bounding box accompanied by a confidence score (Figure 4). The Asbestos Inspection software displayed the detection image, number of detected asbestos fibers, and airborne asbestos concentration (fibers/L) on the screen.

### 3.4. Evaluation of the Automated System for Detecting Airborne Asbestos Fibers

**Figure 5:**
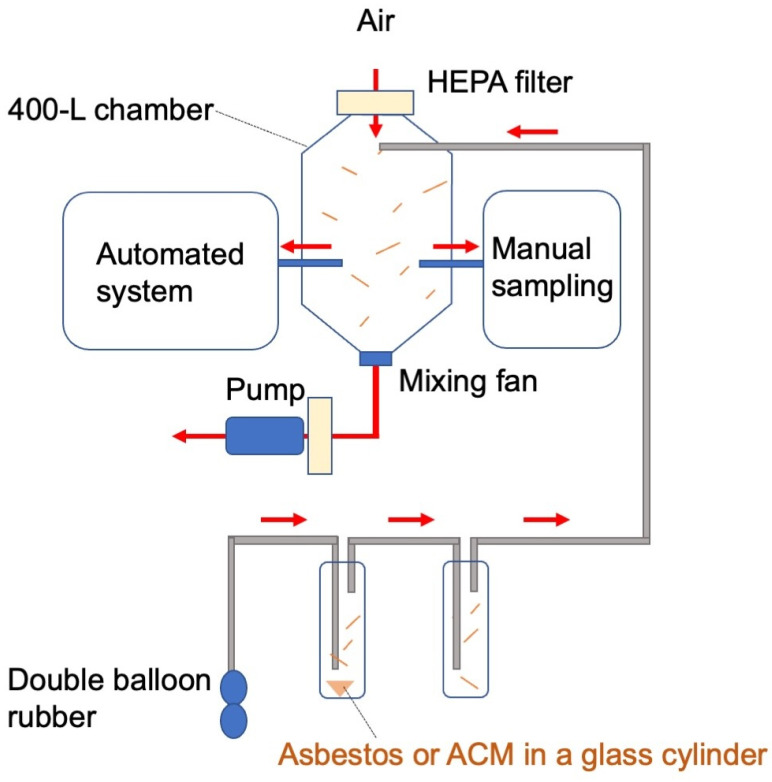
Schematic of the particle dispersion chamber utilized to evaluate the automated system. Airborne particles were generated by dispersing asbestos fibers or pulverized ACM samples within a glass cylinder and subsequently introduced into the 400-L chamber. The inner walls were coated with an antistatic agent to minimize adhesion of asbestos fibers. In addition, the air inside the chamber was continuously mixed using a fan to ensure a homogeneous distribution of airborne particles. Sampling tubes connected to the automated system and manual testing setup were inserted into the chamber for parallel measurements. The red arrows represent the direction of airflow.

**Figure 6:**
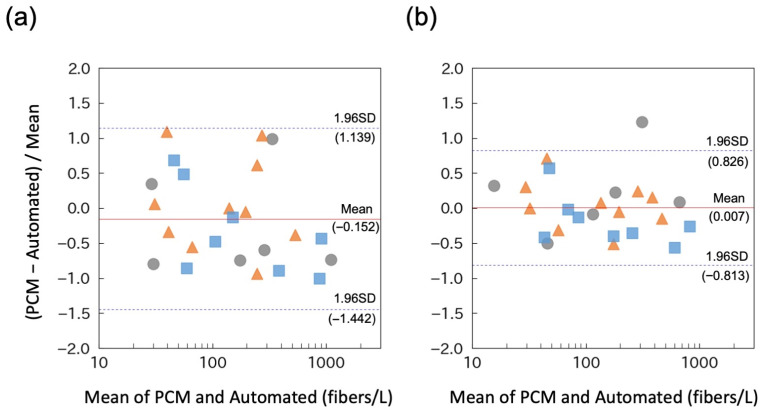
Evaluation of the automated system using standard asbestos samples. (**a**) Relative differences, defined as the difference (PCM—the automated system) divided by the mean of the two measurements, plotted against their mean values (Bland–Altman analysis). (**b**) Images captured at five different positions within a single collection spot, and their averaged values employed for the Bland–Altman analysis. Circles, chrysotile; triangles, amosite; squares, crocidolite. The red solid line and dashed lines indicate the mean difference and the limits of agreement (mean ± 1.96 SD), respectively.

First, standard asbestos dusts (chrysotile, amosite, and crocidolite) were individually introduced into a 400-L chamber (Figure 5). The automated system was connected to the chamber using a sampling tube. For comparison with manual measurements, air samples were collected manually on a 25 mm diameter nitrocellulose membrane filter using a separate sampling tube and pump. These filter samples were subsequently analyzed using PCM for comparison. To evaluate the agreement between the PCM and the automated system, a Bland–Altman analysis was conducted (Figure 6a). Because the magnitude of the differences between the two methods varied with concentration, relative differences (defined as the difference divided by the mean of the two measurements) were employed. The mean relative difference in asbestos concentrations measured using PCM and the automated system was −15.2%, with 95% limits of agreement (mean ± 1.96 SDs) ranging from −144.2% to +113.9%. These results indicate that, on average, the two methods yield broadly comparable results; however, substantial variability was observed at the level of individual measurements.

To minimize this variability, images were captured at five distinct positions within a single collection spot by manually shifting the imaging position in the X–Y direction, and the mean values were used for comparison (Figure 6b). This approach yielded a mean relative difference of +0.7%, with narrower limits of agreement (−81.3% to +82.6%) compared with those obtained from single-position analysis (Figure 6a). These results demonstrate that increasing the number of analyzed positions enhanced agreement with PCM measurements and reduced variability.

The mean relative differences in chrysotile, amosite, and crocidolite were +21.2%, +4.6%, and −19.6%, respectively. These findings suggest that the automated system may detect crocidolite more sensitively than PCM, but not chrysotile or amosite. The underlying cause of this mineral-dependent discrepancy remains unclear and warrants further investigation. Notably, similar variability was observed within PCM counting itself. When two independent counts of 10 fields of view from a single PCM sample (PCM1 and PCM2) were compared, the mean relative difference was −22.1%, with limits of agreement ranging from −104.6% to +60.3% (Supplementary Figure S2). This indicates that substantial variability can inherently arise from random fluctuations in fiber counts between individual fields of view, even when using the conventional reference method.

**Figure 7:**
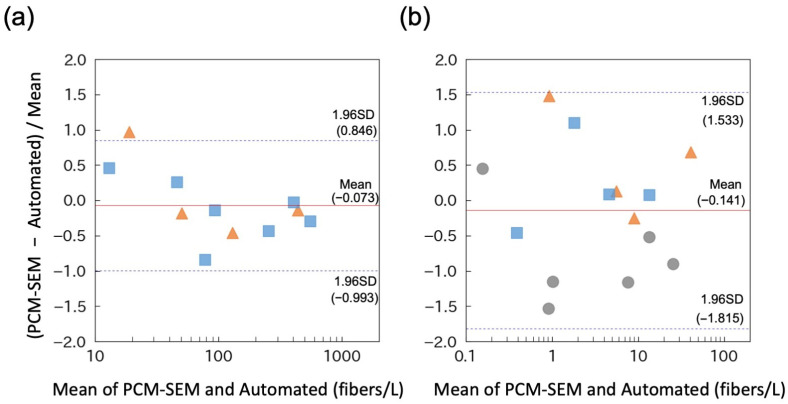
Evaluation of the automated system using ACM samples. (**a**) Pulverized calcium silicate board containing 6.6% amosite, 4.1% crocidolite, and 2.8% chrysotile (squares) as well as calcium silicate board containing 24% amosite and 0.9% chrysotile (triangles) were aerosolized in the chamber. Relative differences between PCM–SEM and the automated system were plotted against their mean values (Bland–Altman analysis). (**b**) Concentration of airborne ACM particles in the chamber reduced using an exhaust pump. To evaluate performance at lower concentrations, sampling times were extended to 30 and 100 min. Pulverized slate boards containing 7.4% chrysotile (circles) were also evaluated. The red solid line and dashed lines indicate the mean difference and the limits of agreement (mean ± 1.96 SD), respectively.

In real-world demolition environments, asbestos fibers are present alongside various non-asbestos fibers and particulate matter. To assess potential interference under these conditions, an asbestos-free calcium silicate board was pulverized and aerosolized in a chamber as a negative control. The automated system detected no fluorescent fibers (Supplementary Figure S3), confirming the specificity of the fluorescent staining procedure under these conditions. Further evaluations were conducted using asbestos-containing calcium silicate and slate boards. In these experiments, images were also captured at five different positions within a single collection spot by manually shifting the imaging position in the X–Y direction, and PCM counts were adjusted based on the proportion of asbestos fibers among the total fibers, as determined through SEM analysis (PCM–SEM). The mean relative difference in asbestos concentrations measured through PCM–SEM and the automated system was −7.3%, with limits of agreement ranging from −99.3% to +84.6% (Figure 7a). These findings demonstrate that the two methods yield broadly comparable results for pulverized ACM samples, with limits of agreement similar to those observed for standard asbestos dust.

**Figure 8:**
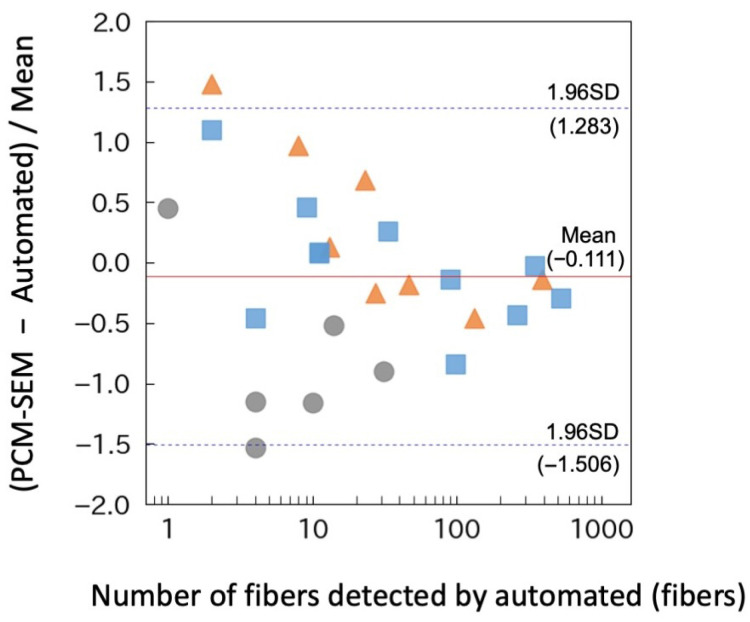
Mean relative difference replotted against the number of fibers detected using the automated system. Data from Figure 7a,b are combined for this analysis. Circles, pulverized slate boards containing 7.4% chrysotile; triangles, pulverized calcium silicate board containing 24% amosite and 0.9% chrysotile; squares, pulverized calcium silicate board containing 6.6% amosite, 4.1% crocidolite, and 2.8% chrysotile. The red solid line and dashed lines indicate the mean difference and the limits of agreement (mean ± 1.96 SD), respectively.

To further examine relative differences at lower asbestos concentrations, pulverized ACM samples were diluted and sampling durations were increased. For example, with a 100-min sampling time, the concentration corresponding to a single detected fiber was calculated as 0.3 fibers/L. Under these conditions, the mean relative difference in asbestos concentrations measured by PCM–SEM and the automated system was −14.1%, with limits of agreement ranging from −181.5% to +153.3% (Figure 7b), indicating considerable dispersion at low asbestos concentrations. To further investigate this variability, the mean relative difference was replotted against the number of fibers detected by the automated system (Figure 8). The analysis revealed that variability increased as the number of detected fibers decreased. This trend is consistent with stochastic counting effects inherent in low-count measurements, where random fluctuations in fiber counts between individual fields of view can amplify the relative differences between the two methods. Therefore, the wide limits of agreement observed at low concentrations likely reflect the counting variability rather than a systematic disagreement between the methods. Collectively, these results demonstrate that the automated system is capable of providing reliable asbestos concentration measurements, particularly when a sufficient number of fibers is detected.

## 4. Discussion

Airborne respirable asbestos fibers present significant health hazards; however, their detection remains complex and time-consuming. Continuous monitoring of asbestos fiber concentrations at demolition sites necessitates practical and efficient solutions. Although fiber monitoring devices utilizing scattered light measurements have been developed [23], these instruments lack specificity for asbestos detection. Similarly, the conventional PCM method is limited by its inability to distinguish asbestos from other fibrous particles without supplementary analysis—typically requiring SEM or TEM [10,11]—which is impractical for on-site use. In contrast, the FM method offers greater selectivity for asbestos fibers compared with the PCM, offering a potential alternative for field-deployable asbestos monitoring [14]. For example, Cho et al. developed a dual-mode, high-throughput microscopy system capable of rapidly acquiring both fluorescence and reflected light images of asbestos sample slides [18]. This system automates image acquisition with fiber enumeration using ImageJ software. However, fully automated airborne asbestos detection based on the FM method still required manual integration of air sampling and fluorescent staining steps prior to imaging [18]. In this study, we have advanced this approach by integrating air sampling, fluorescent staining, and imaging into a seamless and fully automated workflow for continuous PM_2.5_ monitoring. Unlike PCM methods, the FM approach eliminates the need for acetone treatment to render the membrane filter transparent [15], thereby enabling direct compatibility with the roll-based membrane filter mechanism employed in continuous PM_2.5_ monitoring.

The performance of the automated system was evaluated using both standard asbestos samples and airborne fibers originating from ACMs. Results demonstrated that the automated system was generally comparable with conventional PCM and PCM–SEM methods. However, significant variability was observed at the level of individual measurements, particularly at low asbestos concentrations. This variability likely resulted from the stochastic effects associated with the low number of detected fibers, a phenomenon consistent with established counting statistics in fiber measurements, where the relative uncertainty increases as the number of counted fibers decreases (Figure 8). Consistent with this interpretation, capturing images at five distinct positions within a single collection spot and using their mean values improved the agreement with conventional methods and reduced variability (Figure 6). These findings highlight the importance of expanding the detection area to mitigate stochastic variations in fiber detection. At present, a formal limit of quantification (LOQ) has not been fully established because of the variability at low concentrations. These results indicate that the proposed system is more suitable for continuous monitoring and trend detection (e.g., as a screening or leakage-monitoring tool) than for precise quantification based on a single measurement. In this context, repeated measurements and spatial averaging are essential for improving the statistical robustness in future implementations. Currently, the membrane filter advances by approximately 40 mm per sample, positioning the collected area directly beneath the objective lens of the FM unit. Implementing a finer filter advancement mechanism or incorporating automated X–Y stage control within the 15 mm collection spot would facilitate the acquisition of multiple image fields per sampling event. Such enhancements are expected to further improve measurement accuracy and consistency by minimizing stochastic counting effects.

At the current stage, a notable limitation of the system is the potential for false-positive detections. In this study, the automated system identified fluorescent particles—but not fluorescent fibers—in the pulverized asbestos-free materials (Supplementary Figure S3). These particles demonstrated fluorescence attributed to either intrinsic autofluorescence or being a result of staining with the reagent. When the total number of fluorescent particles exceeded 80 per analyzed field, the AI-assisted fiber-counting software occasionally misclassified clusters of contiguous fluorescent particles as individual fibers (Supplementary Figure S4). These observations suggest that false-positive rates increase under high-background conditions. Further refinement of image-segmentation algorithms and/or control of the sampling volume based on real-time dust monitoring (e.g., using a dust sensor) may help reduce such background-dependent false-positive detections.

The probes are highly selective for asbestos and do not bind to man-made fibers, including glass fibers and Rockwool, or to other asbestos substitutes, such as wollastonite. However, in our previous study, we identified several fluorescent non-asbestos fibers in practical samples [15]. These false-positive fibers included surface-coated mineral wool fibers. Surface coatings, particularly when porous or permeable, may result in nonspecific adsorption of the probes and subsequent nonspecific staining of the fibers. The second-largest group of fluorescent non-asbestos fibers was consistent with the organic microfibers. Such fibers may contain or be coated with various natural or artificial fluorescent dyes, making them autofluorescent even in the absence of fluorescent staining. Despite the presence of these intrinsic false-positive fibers, approximately 95% of the fluorescently stained fibers in demolition site samples were correctly identified as asbestos [15]. In addition to these specificity-related limitations, practical implementation for long-term monitoring also requires careful consideration of reagent stability. Future studies should examine fluorescent reagent stability, background signal, and detection performance under elevated temperatures and realistic temperature fluctuation conditions.

Although the system is currently at the prototype stage and requires additional optimization to reduce variability, the present study demonstrates the conceptual feasibility of integrating air sampling, fluorescent staining, and automated image analysis into a continuous monitoring framework. This study represents the first chamber-based demonstration of a fully integrated system that enables the automated detection of airborne asbestos fibers. The proposed system may present practical advantages in environments where repeated measurements are necessary, such as demolition sites and other occupational environments. By minimizing reliance on manual microscopic analysis, this approach can reduce the analytical workload and limit operator exposure. Furthermore, although the present system was developed specifically for airborne asbestos detection, its basic configuration may provide a useful framework for future studies aimed at detecting other hazardous airborne particles using appropriate fluorescent probes. In addition, its compatibility with network-based data management systems could facilitate continuous environmental monitoring. Additional field validation under diverse real-world conditions is essential to evaluate robustness, reproducibility, and long-term operational stability of the system.

## Data Availability

The data generated or analyzed in this study are available from the corresponding authors upon reasonable request.

## Supplementary Materials

The following supporting information can be downloaded at https://www.mdpi.com/article/10.3390/s26103163/s1, Table S1: Confusion matrix of the test results obtained using the Asbestos Inspection software; Figure S1: Control flow of the automated system, including the “Asbestos Host Program,” “Captomator,” and “Asbestos Inspection.”; Figure S2: Bland–Altman analysis using the mean of 10 fields of view (PCM1 and PCM2) from a single PCM sample; Figure S3: PCM (left) and fluorescence image (right) of a pulverized asbestos-free calcium silicate board; Figure S4: Example of misclassification by the AI-assisted fiber counting software.

## Abbreviations

The following abbreviations are used in this manuscript:

PCM	Phase contrast microscopy
FM	Fluorescence microscopy
SEM	Scanning electron microscopy
TEM	Transmission electron microscopy
ACM	Asbestos-containing material
AI	Artificial intelligence
