# Supplementary material for: An Automated Fluorescence Microscopy-Based Sensing System for Continuous Detection of Airborne Asbestos Fibers on a PM2.5 Monitoring Platform
Source: Sensors (Basel). 2026 May 16;26(10):3163. doi: 10.3390/s26103163 (PMC13210768; doi:10.3390/s26103163)
Supplement: Supplementary file 1 [file sensors-26-03163-s001.zip › sensors-4278708-supplementary.pdf]

Supplementary Materials

An Automated Fluorescence Microscopy–Based Sensing System for Continuous Detection of Airborne Asbestos Fibers on a PM2.5 Monitoring Platform

Akio Kuroda[1]*, Kenichiro Kaga[2], Tomoki Nishimura[1], Kyoka Ichikawa[1], Shogo Yamazaki[1], Hisakage Funabashi[1], Takeshi Ikeda[1], and Takenori Ishida[1]

[1] Graduate School of Integrated Sciences for Life, Hiroshima University
1-3-1 Kagamiyama, Higashi-Hiroshima, Hiroshima 739-8530, Japan
[2] DKK-TOA Corporation
613 Kitairiso, Sayama 350-1388, Japan

***Corresponding authors:**
Akio Kuroda
Tel: +81-82-424-7758
Fax: +81-82-424-7047
Email: akuroda@hiroshima-u.ac.jp

Table S1. Confusion matrix of the test results obtained using the Asbestos Inspection software.

		**YOLOv4 predicted**	
		**Asbestos**	**Non-asbestos**
Manually counted (NIOSH7400)	Asbestos	101	11
	Non-asbestos	11	2446
Accuracy		0.991	
Recall		0.902	
Precision		0.902	
F1-score		0.902	

Asbestos fibers (≥5 μm in length and <3 μm in diameter) were manually counted according to the National Institute for Occupational Safety and Health (NIOSH) Method 7400 [9]. Accuracy, recall, precision, and the F1-score were calculated as previously described [17].

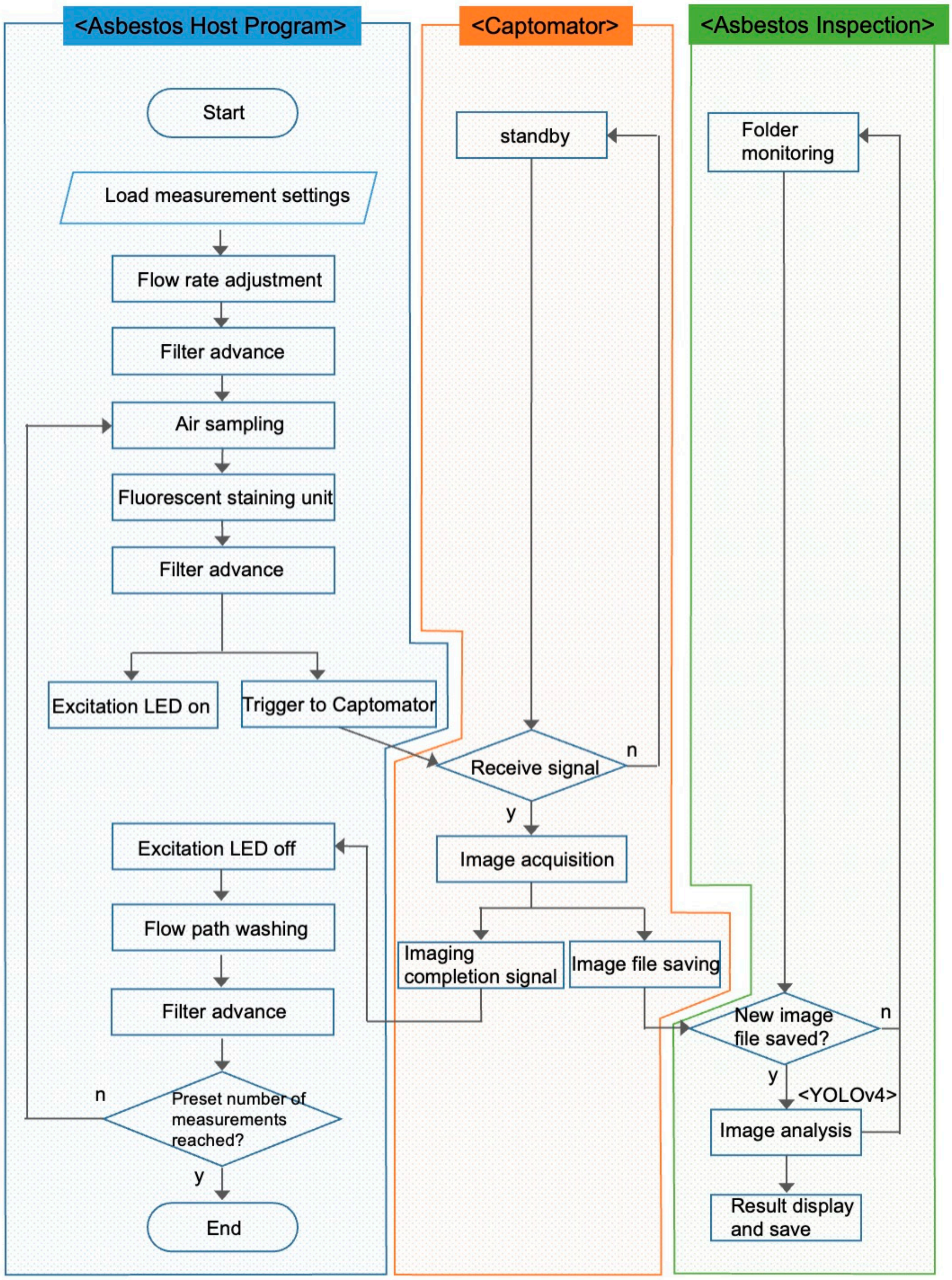

Figure S1. Control flow of the automated system, including the "Asbestos Host Program," "Captomator," and "Asbestos Inspection." The Asbestos Host Program controls the entire measurement sequence by

regulating airflow, advancing the membrane filter, performing automated staining via tube pumps, triggering image acquisition through an external controller (Captomator software, Mitani Corporation, Tokyo, Japan), and repeating the measurement cycle until the preset number of measurements is reached. The Asbestos Inspection software includes AI-assisted asbestos recognition and fiber counting based on the YOLOv4 deep-learning model. The program monitors a designated folder where focus-stacked images are saved and automatically analyzes the images to detect asbestos fibers. It displays the detection results together with the processed image, the number of detected fibers, and the calculated airborne asbestos concentration (fibers/L).

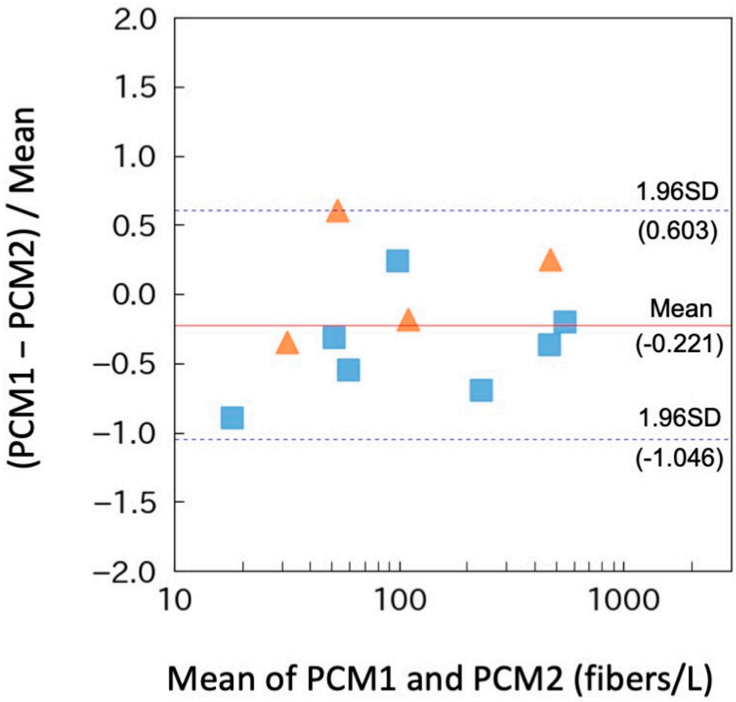

Figure S2. Bland–Altman analysis using the mean of 10 fields of view (PCM1 and PCM2) from a single PCM sample. Pulverized calcium silicate board containing 6.6% amosite, 4.1% crocidolite, and 2.8% chrysotile (squares) as well as calcium silicate board containing 24% amosite and 0.9% chrysotile (triangles) were aerosolized in the 400-L chamber. Air samples were manually collected on a 25 mm diameter nitrocellulose membrane filter using a sampling tube and pump, and the filters were analyzed by PCM. Fibers in each of the 10 fields of view from a single PCM sample were counted independently (PCM1 and PCM2). Relative differences between PCM1 and PCM2 were plotted against their mean values using Bland–Altman analysis. This analysis illustrates the inherent counting variability in PCM measurements arising from random fluctuations in fiber counts between individual fields of view.

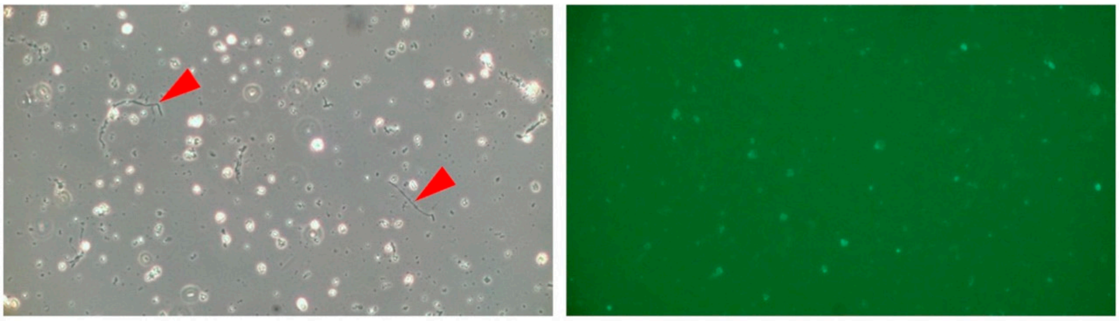

Figure S3. PCM (left) and fluorescence image (right) of a pulverized asbestos-free calcium silicate board. An asbestos-free calcium silicate board was pulverized and aerosolized in a chamber as a negative control. Fibers detected under PCM (red arrows) showed no fluorescent signals under the FM system. The fluorescent particles were observed either as a result of autofluorescence of the particles or fluorescence generated by staining with the reagent.

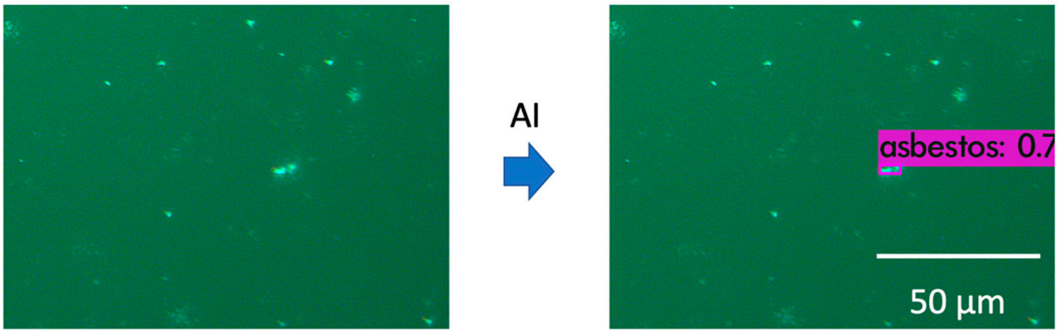

Figure S4. Example of misidentification by the AI-assisted fiber counting software. In some cases, contiguous fluorescent particles were incorrectly recognized as individual fibers.
